# Bone mineral content determined by energy X-ray absorptiometry correlates with handgrip strength in Latin American divers

**DOI:** 10.3389/fpubh.2025.1591242

**Published:** 2025-07-14

**Authors:** Alex Véliz, Raquel Pereira, Anita Dörner, Cristian Álvarez

**Affiliations:** ^1^Departamento de Ciencias Sociales, Universidad de Los Lagos, Puerto Montt, Chile; ^2^Departamento de Ciencias de La Actividad Física, Universidad de Los Lagos, Puerto Montt, Chile; ^3^Departamento de Salud, Universidad de Los Lagos, Puerto Montt, Chile; ^4^Exercise and Rehabilitation Sciences Institute, School of Physical Therapy, Faculty of Rehabilitation Sciences, Universidad Andres Bello, Santiago, Chile

**Keywords:** bone mineral density, blood pressure, cardiometabolic risk, diver, body composition, fat-free mass, handgrip, dual X ray absorptiometry

## Abstract

**Introduction:**

Although a lack of gravitational stimuli decreases bone mineral content in some populations, such as astronauts’ bone mineral content (BMC), little is known about the association of BMC with handgrip muscle strength (HGS) in divers of different years of diving experience.

**Objectives:**

To describe the BMC and body composition of diver workers of different years of diving experience. A second objective was to associate the level of BMC with HGS.

**Materials and methods:**

A descriptive and longitudinal pilot study was carried out in adult men divers of different years of diving experience; tertile 1 of diving experience 1–20 years (T1DE, *n* = 27), tertile 2 of diving experience 21–35 years (T2DE, *n* = 11), and tertile 3 of diving experience 36–45 years (T3DE, *n* = 17). Primary variables were BMC, total fat-free mass (FFM), and total body fat mass (BF), which were analyzed using dual X-ray absorptiometry (iDXA) equipment and the average of both arms’ handgrip strength (HGS_av_). Secondary variables were lifestyle patterns, anthropometry, and other socio-demographic variables.

**Results:**

T3DE showed significant differences *vs*. T1DE group in BMC_Legs_ (*diff*. −107.9 g, *p* = 0.029), BMC_RL_ (*diff*. −51.4 g, *p* = 0.039), and BMC_LL_ (*diff*. −55.7 g, *p* = 0.037). T3DE showed significant differences *vs*. T1DE group in outcomes total FFM (*diff*. −5011.9 g, *p* = 0.015), FFM_Arms_ (*diff*. −1275.1 g, *p* = 0.009), FFM_RA_ (*diff*. −472.3 g, *p* = 0.012), FFM_LA_ (*diff*. −406.6 g, *p* = 0.028), FFM_Legs_ (*diff*. −2117.8 g, *p* = 0.031), FFM_RL_ (*diff*. −1046.3 g, *p* = 0.037), and FFM_LL_ (*diff*. −1071.3 g, *p* = 0.031). There was a significant correlation between HGS_av_ and total BMC (*R*^2^ = 21.3%), between HGS_RA_ and total BMC (*R*^2^ = 21.1%), between HGS_LA_ and total BMC (*R*^2^ = 20.2%), between HGS_av_ and BMC_Arms_ (*R*^2^ = 28.8%), between HGS_RA_ and BMC_Arms_ (*R*^2^ = 27.9%), between HGS_LA_ and BMC_Arms_ (*R*^2^ = 27.8%), between HGS_av_ and BMC_Legs_ (*R*^2^ = 13.3%), between HGS_RA_ and BMC_Legs_ (*R*^2^ = 13.8%), and between HGS_LA_ and BMC_Legs_ (*R*^2^ = 11.9%).

**Conclusion:**

Diver workers with greater years of experience exhibit a reduced level of BMC and FFM in the legs compared to peers with less experience, and HGS is associated with BMC. Future longitudinal studies in diver workers could explore BMC and body composition in these populations to confirm our findings and include other physical fitness parameters in different diving specialties.

## Introduction

The worldwide prevalence of the most common bone diseases, such as osteoporosis and osteopenia, has been increasing in recent decades, particularly in the adult and older adult populations (1, 2). The World Health Organization (WHO) defines osteoporosis as a criterion defined as a T-score of at least −2.5 standard deviations (SD) at the femoral head or lumbar spine compared to young people in the same population, with a lower score of −1.0 to −2.5 SD using bone mineral content (BMC) (3), being a low BMC of (43.1%), a precursor to a diagnosis of osteoporosis (2).

In the United States, approximately 12% of the population over 50 years of age has osteoporosis, with a higher prevalence in women (19.6%) compared to men (4.4%) (2). Other epidemiological evidence from a recent cross-sectional study based on the National Health Survey of Chile revealed a prevalence of 10.9% of frailty and 59% of pre-frailty, with women representing 64.9% and men 35.1% (i.e., frailty as defined by Fried’s Phenotype Scale) (4). There are few studies in Chile about BMC and the prevalence of osteoporosis in divers, and there is an important need to increase studies in body composition analyses to favor a major policy of preventing bone diseases. Although hydrodensitometry and air displacement plethysmography are valid methods, in parallel, computed tomography with magnetic resonance imaging are considered gold standard methods for body composition analysis (particularly for fat depots) (5). However, for BMC estimation, dual-energy X-ray absorptiometry (iDXA) is also considered a gold standard, with minimum radiation exposure (5–7). Previous studies by iDXA analyses reported that low BMC is consecutively associated with an increased risk of frailty in the adult population (8).

Several outcomes associated with physical activity, such as muscle strength, stimulate bone formation and preserve BMC in adult males (9). Briefly, tennis and squash players show higher BMC levels in the upper limbs and upper extremities compared to non-athlete controls, showing a bone-specific adaptation from the skeleton/muscle behavior (10). The same applies to other body segments, in which endurance athletes (i.e., that regularly are walking and running) in comparison to those trained in muscle strength (i.e., weightlifting) the latter have a higher BMC about endurance athletes, being recommended to population with lower BMC to practice more exercise/sport of muscle strength to improve their bone mineralization (11). Conversely, other factors are related to a reduction in BMC, such as diseases like cancer (12), environmental pollution (13), sleep disturbance (14), or the conditions of sedentarism or physical inactivity, where both situations can worsen or improve the BMC process (15, 16). Other factors, such as a diet rich in ultra˗processed foods (1), menopause, and aging, can also contribute significantly to accelerating the reduction of BMC. Considering this information and the need to predict frailty and BMC deterioration, some physical fitness outcomes are associated with the BMC, such as energy expenditure (17) and muscle size (18, 19). However, there is little knowledge about other physical fitness outcomes, such as handgrip muscle strength (HGS), for predicting BMC in adult workers.

Far from athletes or the sedentary population, there are jobs with specific environmental conditions that can put health maintenance at risk, such as diving. Diver fishermen (i.e., divers who live on the Chilean coast littoral) have behavioral and lifestyle characteristics that modulate their musculoskeletal health. Diving reduces the gravitational environment under the water, similar to sedentary lifestyle actions, or lying in bed, or space (20). Since the viscosity of water is greater than that of air, diving creates a significant load, raising the pressure by 1 atmosphere (atm) for every 10 m of depth. Therefore, diving provides a low-gravity environment, and while it requires repetitive muscular movements (i.e., in the context of the diver fisherman work), these actions do not impose a substantial impact load on the human skeleton, potentially affecting BMC (21) and leading to changes in gut microbiota (22). Conversely, muscular movement in the human skeleton, through muscle strength exercise, is considered a potent osteogenic stimulus (23). A recent governmental study conducted by the Chilean Superintendence of Social Security (SUSESO) showed that 36% of divers in the aquaculture and fishing industry sector report a smoking habit, while a concerning 76% admit to occasional alcohol consumption. Moreover, bone fractures (~4 to 5%) are the primary type of accidents reported (24). Furthermore, they have a high prevalence of overweight and obesity (86.7%, almost double the national Chilean prevalence), which further increases their cardiovascular risk (24). These lifestyle conditions and the physiological demands of diving underscore the need for increased research in physiological adaptations to body composition and occupational areas.

Moreover, epidemiological studies in Chile have shown an increase in the prevalence of cardiometabolic diseases, including hypertension, diabetes, and metabolic syndrome in the adult population, with a significant prevalence in people over 65 years (25–27). Furthermore, considering the importance of preventing osteoporosis and low BMC in the adult population, the abundance of risk factors for preserving the skeletal muscle mass (i.e., body composition), there is a need to add more knowledge about other easy-to-measure outcomes for predicting BMC and body composition, such as HGS. Thus, due to physiological diving conditions promote an environment of high risk in the vascular system, and considering the current unhealthy lifestyle of diver workers, there is a particular need to ask the questions whether HGS show relationship with BMC, muscle mass or fat free mass in diver workers and to clarify if the accumulation of more years of experience working on diving (i.e., under a low gravitational work) play a role in the total or segmental BMC deterioration. In solving these questions, HGS could be used to monitor bone health and the body composition risk when muscle mass declines in these populations. Thus, the objective of the present study was to describe the BMC and body composition of diver workers of different years of diving experience. A second objective was to associate the level of BMC with HGS. A second objective was to associate the level of HGS with BMC. We hypothesized that HGS is significantly associated with BMC in Chilean diver workers.

## Materials and methods

### Population and study design

A pilot, descriptive and longitudinal type study developed in the laboratory of the Universidad de Los Lagos, in the city of Puerto Montt, Chile, between June and September 2024, with diver workers from diving social groups of the cities Puerto Montt, Calbuco, Maullín and Ancud, all corresponding to the geographical southern macro-zone coast of Chile. The participants were contacted through a broad call through social networks or by directly contacting the leaders of each organization. Each group was explained by the objectives of the study, its scope, and the processes for delivering evaluations and counseling in this regard.

**Figure 1:**
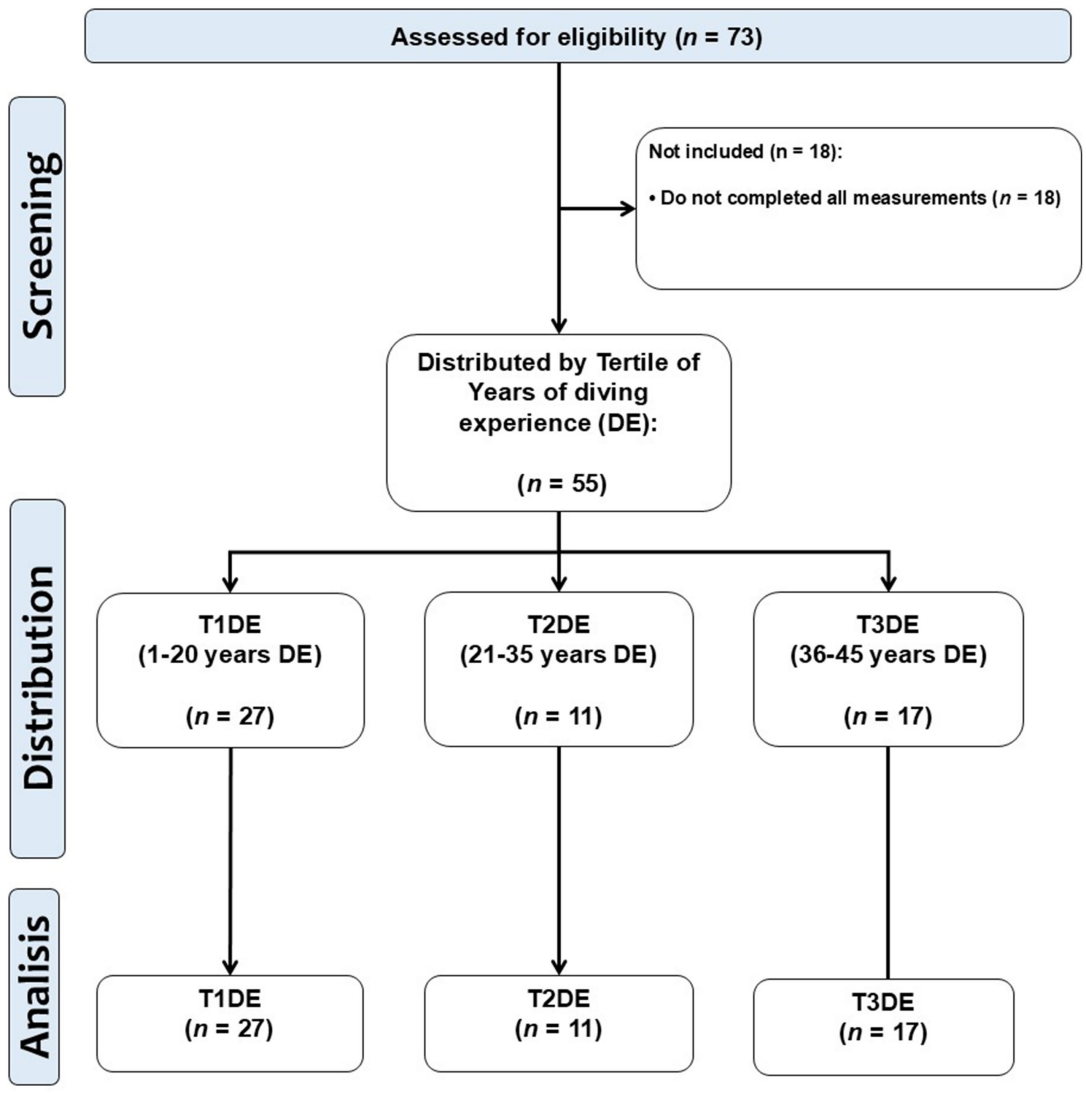
Study design.

The study was approved by the Scientific Ethical Committee of Scientific Ethical Committee of Universidad Mayor, by the approval folio N° 0492. All participants had previously signed an informed consent form indicating the procedures to be performed, protection of personal data, anonymity, potential risks, among other aspects. The sample was calculated using a similar adult male population based on standard deviation (SD 8.6 kg) (28). Thus, looking for detecting associations and a moderate effect size, a sample size of (*n* = 55) participants provides a statistical power of 80% in correlation/regression analyses. This calculation was developed using the freely available G*Power software (version 3.1.9.6, Franz Faul, Universität Kiel, Kiel, Germany). The final sample included groups of diver workers of different tertiles of years of diving experience, and was (*n* = 55) distributed by the following tertiles; tertile 1 of years of diving experience 1–20 years (T1DE, *n* = 27), tertile 2 of years of diving experience 21–35 years (T2DE, *n* = 11), and tertile 3 of years of diving experience 36–45 years (T1DE, *n* = 17). The study design can be seen in Figure 1.

### Bone mineral content by dual X ray absorptiometry analysis (main outcomes)

**Figure 2:**
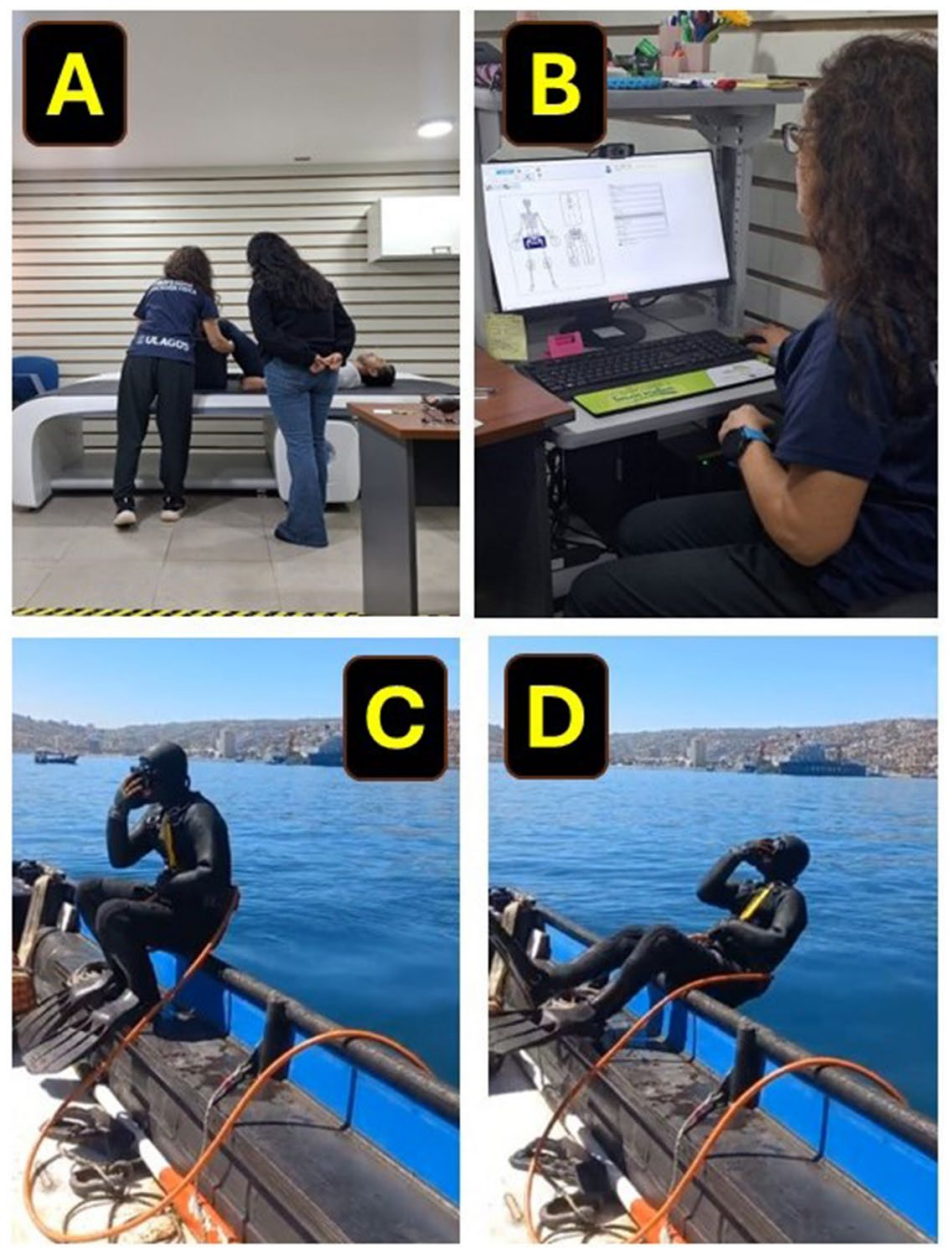
Dual X absorptiometry measurement in Latin American diver workers from the coast of Chile. **(A)** Show the preparation phase of the divers for the iDXA measurement. **(B)** Show the evaluator scanning BMC and body composition in the computer software. **(C)** Show the characteristic equipment in which diver workers operate on the Chilean coast, and **(D)** show the position where the divers usually contact the seawater.

The subjects attended the Universidad de Los Lagos laboratory for the body composition evaluation from Monday to Friday from 9:00 to 13:00 in the morning for these procedures. Before the measurement of body composition with the iDXA equipment (Healthcare General Electric Company, ENCORE 18 Software, United States), a preliminary interview was conducted to rule out the use of electronic devices such as pacemakers, insulin pumps, among others that could be declared and interrupt the operation of the equipment or could affect the health of each participant. For the iDXA measurement, each subject was placed in a supine position on the stretcher of the equipment, wearing light clothing and without shoes or metal objects. The evaluation process lasted an average of 10 min, but each subject participating was inside the evaluation room for 20 min. The iDXA measurements and the characteristics of the diver’s work can be seen from (Figures 2A–C).

### Lifestyle patterns

The physical activity (PA) levels of the population were determined with the international physical activity questionnaire “Global Physical Activity Questionnaire” (GPAQ v2) (29). From here, the min/week of vigorous (PAVI), moderate (PAMI), and light (PALI) was determined and registered by each diver participant. The sedentary time per week was determined by self-reporting time spent on activities involving sitting or reclining during leisure time.

To determine the variables related to smoking habits, questions were considered based on the minimum smoking surveillance instrument used by the Pan-American Health Organization. These identified the current smokers (daily and occasionally) and ex-smokers, allowing for the number of cigarettes smoked and the persistence of the habit; however, in this study, the information was registered only as non-smokers and smokers (30).

### Handgrip strength (main outcomes)

For HGS, measurements of both hands were used in three attempts in a seated position, and we used the average of both arms (HGS_av_), handgrip of the right (HGS_RA_: HGS right arm), and left (HGS_LA_: HGS left arm) arm to be registered. These measurements were developed by using a digital handheld dynamometer (Jamar®, PLUS+, Sammons Preston, Patterson Medical, Illinois, United States) following previous studies in Latin American populations (31).

### Anthropometry (secondary variables)

Weight was measured with a BIA equipment InBody120™ scale (tetrapolar 8-point tactile electrode system, model BPM040S12F07, Biospace, Inc., Seoul, Korea) with a 0.1 kg precision, following previous studies (32). Height was measured with a SECA™ 213-Topmedic portable stadiometer (Germany). BMI was calculated using weight and height squared.

### Blood pressure

Blood pressure was measured on three attempts with at least 1 min rest intervals between measurements, using a digital cuff instrument positioned in the arm OMRON™ (Model HEM-7142, United States). Blood pressure was categorized according to the criteria of the European Society of Cardiology into hypertension (systolic blood pressure [SBP] ≥ 140 or diastolic blood pressure [DBP] ≥ 90 mmHg), normal-high blood pressure (SBP 130–139 mmHg or DBP 85–89 mmHg) (33).

### Ruffier test

All the subjects evaluated were instructed in the following way: firstly, the heart rate at rest was measured in a standing position for 15 s multiplied by 4 to know the equivalent beats per minute, recorded as (HR1), then the person stood up, did leg flexion-extensions at a steady pace with the throne straight at 90° of knee flexion, raising arms in front of the front while flexing for 45 s. Immediately after performing the leg flexion-extensions exercise, the heart rate (HR2) is recorded again, followed by a 1-min rest in a seated position. Finally, the heart rate (HR3) is recorded again. The results are then interpreted using the following formula, Ruffier index: [(HR1 + HR2 + HR3)–200]/100, where the aerobic endurance is determined according to the following values [0 very good performance, 0.1–5 good performance, 5.1–10 average performance, >10–15 insufficient performance, 15.1–20 poor performance] (requires medical evaluation).

### Statistical analysis

Data are shown as mean and (±) standard deviation for continuous variables and as frequency (*n* =) and percentage (%) for categorical variables. The sample was divided into tertiles of years of diving experience (T1DE, T2DE, and T3DE). The Shapiro–Wilk test was applied to test the normal distribution of the main and secondary outcomes (See Supplementary material https://figshare.com/s/b6f46fecbb2a71e0fc23). To test differences between groups, One-way ANOVA and Tukey’s *post hoc* test for multiple comparisons between groups at *p* < 0.05 alpha error level were applied for outcomes with normal distribution. Furthermore, Cohen’s *d* effect size test was reported at the *p* < 0.05 level for this interaction. Conversely, the Kruskal-Wallis test was applied to outcomes with no normal distribution, with Dunn’s *post hoc* test for multiple comparisons at *p* < 0.05 alpha error level. To the secondary outcome of physical activity of vigorous intensity (PAVI), the mixed-effects model (REML) was applied with the Holm-Sidak test for multiple comparisons. Simple linear regression was applied to test the association between total BMC, arm, and leg BMC with the variables HGS_av_ and HGS of the right (HGS_RA_) and left arm (HGS_LA_). The level of prediction of BMC was recorded using the R^2^ determination coefficient, and the predictive equation was reported. The analyses used GraphPad Prism v. 8.0 statistical software (Chicago, Illinois, United States).

## Results

The results indicate that there were significant differences among T1DE, T2DE and T3DE in the variable age (T1DE 46.1 ± 2.2; T2DE 56.3 ± 0.5 and T3DE 64.2 ± 1.6 years, *p* < 0.0001) (Table 1), years of diving experience (T1DE 19.8 ± 1.7; T2DE 34.8 ± 0.3 and T3DE 41.5 ± 0.7 years of diving experience, *p* < 0.0001) (Table 1). In the anthropometry, it was observed that height was significantly higher in the T2DE vs. T3DE group (173.0 ± 2.0 *vs*. 165.0 ± 2.0 cm, *p* = 0.016), while weight and BMI showed no significant differences (Table 1). Regarding cardiovascular parameters, systolic, diastolic blood pressure, and heart rate at rest did not show significant differences among T1DE, T2DE, and T3DE (Table 1). On the other hand, basal metabolism showed a decreasing trend with more years of diving experience, being significantly different between T3DE *vs*. T1DE (1403.0 ± 90.0 *vs*. 1468.0 ± 89.0 Kcal/kg) and between T3DE *vs*. T2DE (1403.0 ± 90.0 *vs*. 1463.0 ± 151.0 Kcal/kg) (Table 1).

**Table 1:** Cardiovascular, anthropometric, and behavioral characteristics in three different groups of diver workers of different years of diving experience.

Outcomes	Grupos			Between-group interaction
	T1DE^a^	T2DE^b^	T3DE	
(*n* =)	27	11	17	
Age (y)	46.1 ± 2.2	56.3 ± 0.5	64.2 ± 1.6^a^	***p* < 0.0001** ^**&**^
Diving experience (y)	19.8 ± 1.7	34.8 ± 0.3^a^	41.5 ± 0.7^a^	***p* < 0.0001** ^**&**^
Anthropometric				
Height (m)	171.0 ± 2.0	173.0 ± 2.0	165.0 ± 2.0^a,b^	***F***_**(4.4)**_ **; *p* = 0.016** ^**†**^ **; *d* 0.14**
Weight (kg)	84.8 ± 2.7	86.5 ± 3.7	80.2 ± 2.8	*F*_(0.9)_; *p* = 0.384^†^; *d* 0.03
Body mass index (kg·m^2^)	29.0 ± 0.9	28.9 ± 1.2	29.4 ± 1.0	*F*_(0.0)_; *p* = 0.941^†^; *d* 0.002
Normal weight	4 (14.8)	2 (18.2)	2 (11.8)	
Overweight	13 (48.1)	5 (45.5)	8 (47.1)	
Obesity I	7 (25.9)	3 (27.3)	4 (23.5)	
Obesity II	2 (7.4)	1 (9.1)	3 (17.6)	
Obesity III	1 (3.7)	0 (0)	0 (0)	
Arterial hypertension				
Systolic blood pressure (mmHg)	139.3 ± 3.2	141.4 ± 4.3	147.1 ± 4.2	*F*_(1.1)_; *p* = 0.314^†^; *d* 0.04
Diastolic blood pressure (mmHg)	86.8 ± 2.2	86.8 ± 3.3	89.9 ± 3.5	*F*_(0.3)_; *p* = 0.703^†^; *d* 0.01
Heart rate rest (beats/min)	70.0 ± 1.7	61.8 ± 2.2	69.3 ± 3.2	*p* = 0.066^&^
Basal metabolic rate (kcal/kg)	1468.0 ± 89.0	1463.0 ± 151.0	1403.0 ± 90.0^a,b^	***p* = 0.008** ^**&**^
Physical fitness condition				
Ruffier test				
*Ruffier index*	3.21 ± 0.53	1.61 ± 0.68	2.59 ± 0.61	***p* = 0.008** ^**¥**^
Excellent, *n*= / (%)	3 (11.1)	3 (27.3)	2 (11.8)	
Good, *n*= / (%)	19 (70.4)	7 (63.6)	11 (64.7)	
Middle, *n*= / (%)	5 (18.5)	1 (9.1)	4 (23.5)	
Middle-low, *n*= / (%)	0 (0)	0 (0)	0 (0)	
Insufficient, *n*= / (%)	0 (0)	0 (0)	0 (0)	
PA patterns (GPAQ)				
PAVI (d/week)	6026.7 ± 1189.8	7069.1 ± 1771.4	6508.2 ± 1883.8	*p* = 0.870^&^
PAMI (d/week)	2235.6 ± 381.7	2496.4 ± 899.2	3007.1 ± 573.2	*p* = 0.486^&^
PALI (d/week)	2710.9 ± 525.3	3315.0 ± 692.4	4505.5 ± 999.1	*p* = 0.303^&^
Total PA (min/wk)				*p* = 0.434^&^
Total PA (MET/wk)	10973.0 ± 1,596	12880.5 ± 2253.5	12903.1 ± 991.1	*p* = 0.676^&^
Total sedentary time (min/wk)	1470.0 ± 153	1069.1 ± 103.9	1568.8 ± 92.0^b^	***p* = 0.037** ^**&**^
HGS_av_ (kg)	44.5 ± 1.9	42.9 ± 1.3	36.8 ± 1.5^a^	***p* = 0.008** ^**&**^
Smoking habit				
Yes, *n*= / (%)	2 (3.6)	1 (1.8)	1 (1.8)	
No, *n*= / (%)	25 (45.5)	10 (18.2)	16 (29.1)	
Alcohol consumption				
Yes, *n*= / (%)	25 (45.5)	11 (20.0)	17 (30.9)	
No, *n*= / (%)	2 (3.6)	0 (0)	0 (0)	

Data are shown as mean and standard error. Groups are described as: (T1DE) Tertile 1 of diving experience (1–20 years diving), (T2DE) Tertile 2 of diving experience (21–35 years diving), and (T3DE) Tertile 3 of diving experience (36–45 years diving). Outcomes are described as: (HGSav) Handgrip muscle strength average of both arms. (PA) Physical activity. (GPAQ) Global physical activity questionnaire. (PAVI) Physical activity of vigorous intensity (PAMI), Physical activity of moderate intensity (PALI), Physical activity of light intensity. (MET) Metabolic equivalent of task in resting. (†) Data analyzed by One-way ANOVA and Tukey’s post hoc for multiple comparisons among groups at *p* < 0.05 alpha error. (&) Data analyzed by the Kruskal-Wallis nonparametric test and the Dunn’s test for multiple comparisons among groups at *p* < 0.05 alpha error. (¥) Data analyzed by mixed-effects model (REML) and Dunn’s test for multiple comparisons among groups at *p* < 0.05 alpha error. (d) Denotes Cohen’s d effect size at *p* < 0.05. (a) Denotes significantly different versus the T1DE group at *p* ≤ 0.05. (b) Denotes significantly different versus the T2DE group at *p* ≤ 0.05. Bold values denote significant differences among groups.

In terms of cardiovascular recovery after physical effort, the Ruffier index showed better values in the T2DE group, where a higher proportion of divers classified as “excellent” (27.3%) in their cardiovascular recovery was observed compared to the T1DE (11.1%) and T3DE (11.8%) groups (*p* = 0.008) (Table 1). The total sedentary time per week differed between T3DE *vs*. T2DE (1568.8 ± 92.0 *vs*. 1069.1 ± 103.9 min/week, *p* = 0.037) (Table 1). The HGS_av_ decreased with years of diving experience, being significantly lower in the T3DE *vs*. T1DE (36.8 ± 1.5 *vs*. 44.5 ± 1.9 kg, *p* = 0.008) compared to the T1DE and T2DE group (*p* = 0.008) (Table 1). The levels of the different types of physical activity intensities per week did not show significant differences between groups (Table 1). About lifestyle habits, alcohol consumption was high in all groups, being higher in T1DE (93.6%), while smoking was reported by a small percentage of the divers, with no significant differences between groups.

### Bone mineral content

**Figure 3:**
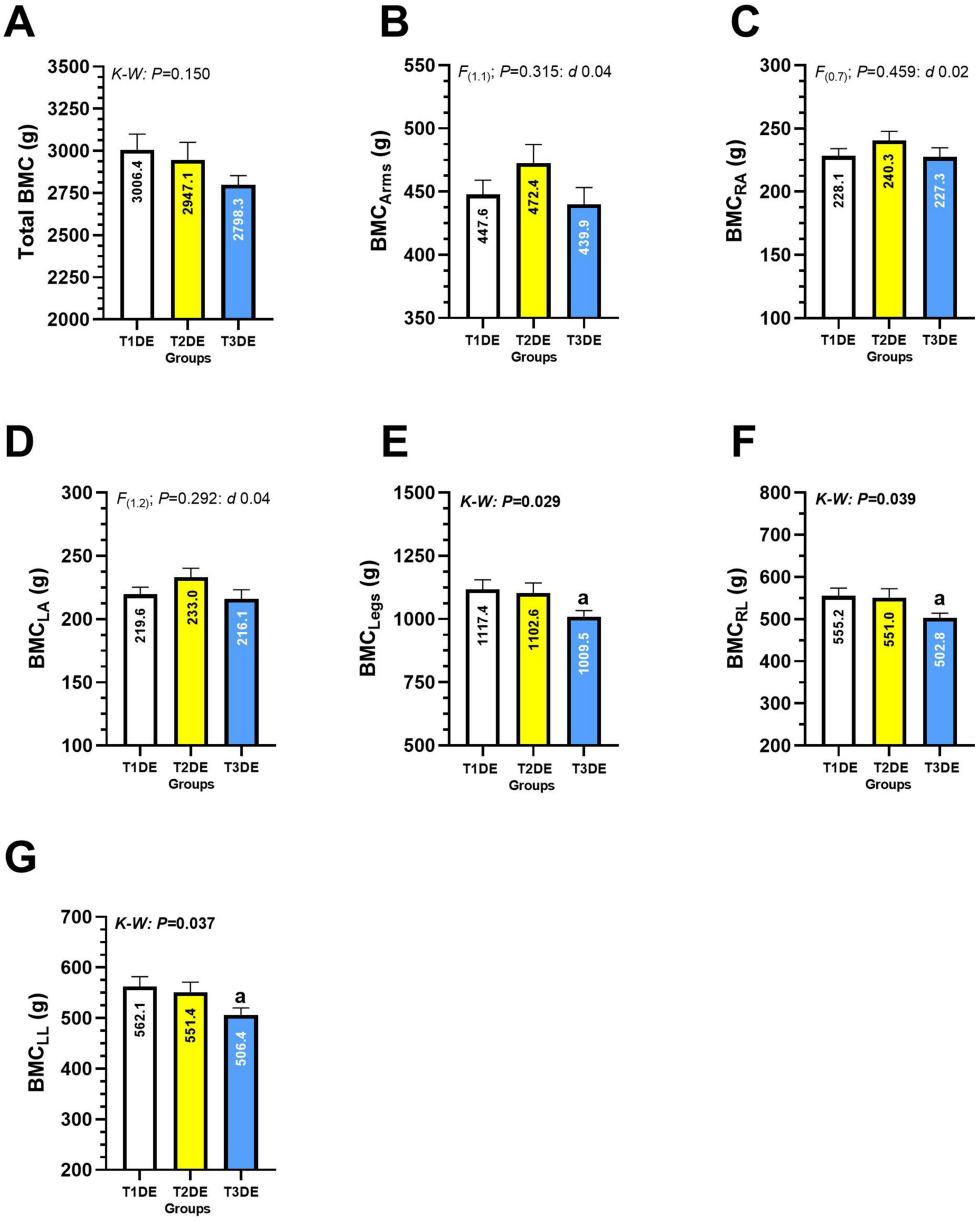
Total bone mineral content **(A)**, of the arms **(B)**, right arm **(C)**, left arm **(D)**, legs **(E)**, right leg **(F)**, and bone mineral content of the left leg **(G)** in Latin American diving workers. Groups are described as: (T1DE) Tertile 1 of diving experience (1–20 years diving), (T2DE) Tertile 2 of diving experience (21–35 years diving), and (T3DE) Tertile 3 of diving experience (36–45 years diving). Outcomes are described as: (BMC_Arms_) Bone mineral content of the arms, (BMC_RA_) Bone mineral content of the right arm, (BMC_LA_) Bone mineral content of the left arm, (BMC_Legs_) Bone mineral content of the legs, (BMC_RL_) Bone mineral content of the right leg, (BMC_LL_) Bone mineral content of the left leg. (K-W) Denotes data analyzed by nonparametric Kruskal-Wallis test at *p* < 0.05 level. (a) Denotes significant differences between T3DE vs. T1DE group at *p* < 0.05 level.

When comparing total BMC, BMC of both arms, and BMC of left and right arm among T1DE, T2DE, and T3DE groups, no significant differences between groups were detected (Figures 3A–D). Conversely, there were significant differences between groups T3DE *vs*. T1DE in outcomes BMC of the legs (1009.5 ± 101.0 *vs*. 1117.4 ± 197.4 g, *p* = 0.029) (Figure 3E), BMC of the right leg (503.8 ± 47.1 *vs*. 555.2 ± 96.6 g, *p* = 0.039) (Figure 3F), and BMC of the left leg (506.4 ± 56.5 *vs*. 562.1 ± 101.9 g, *p* = 0.037) (Figure 3G).

**Figure 4:**
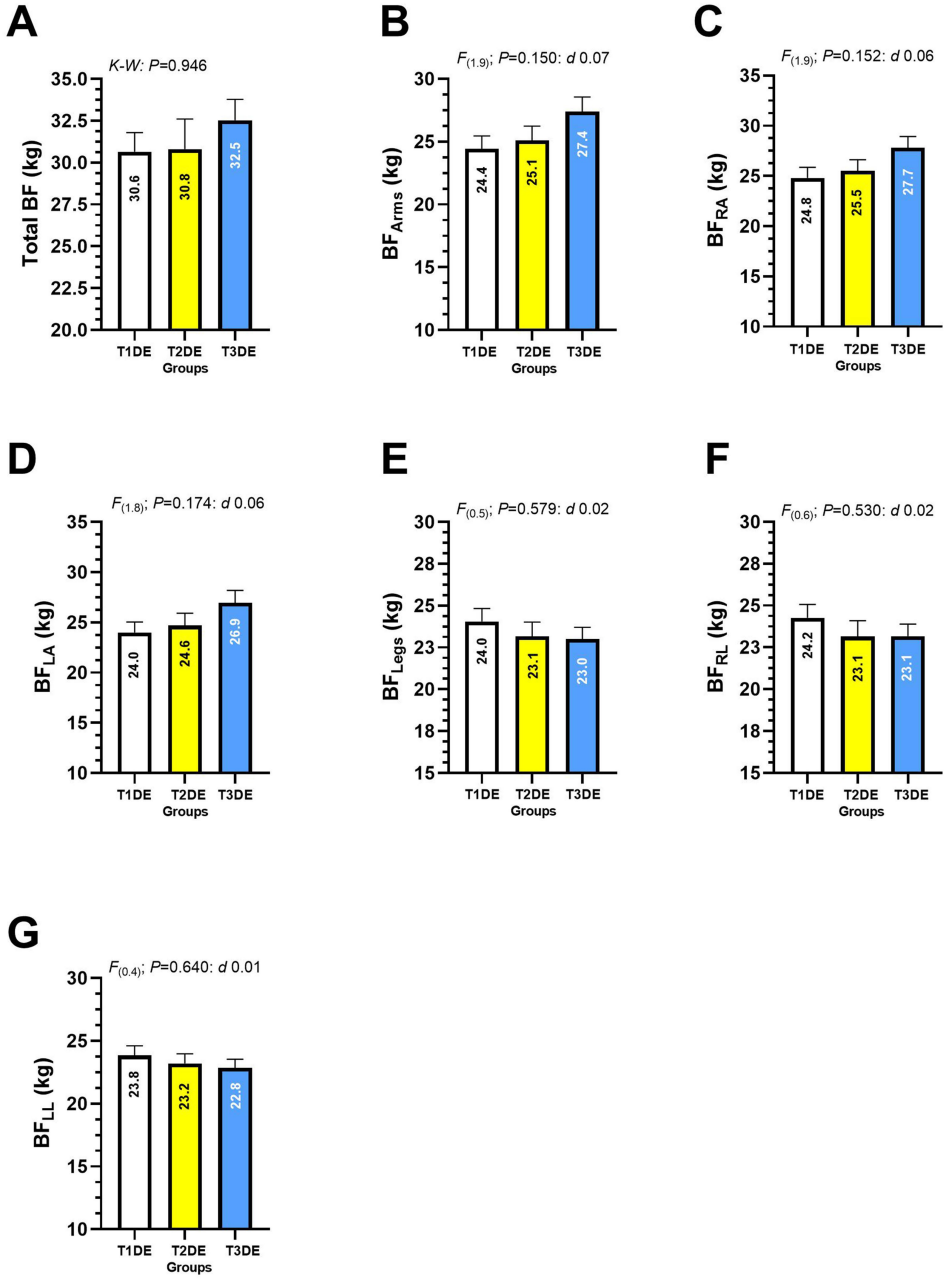
Total body fat **(A)**, body fat of the arms **(B)**, right arm **(C)**, left arm **(D)**, legs **(E)**, right leg **(F)**, and body fat of the left leg **(G)** in Latin American diving workers. Groups are described as: (T1DE) Tertile 1 of diving experience (1–20 years diving), (T2DE) Tertile 2 of diving experience (21–35 years diving), and (T3DE) Tertile 3 of diving experience (36–45 years diving). (BF) Body fat. Outcomes are described as: (BF_Arms_) Body fat of the arms, (BF_RA_) Body fat of the right arm, (BF_LA_) Body fat of the left arm, BF_Legs_ Body fat of the legs, (BF_RL_) Body fat of the right leg, (BF_LL_) Body fat of the left leg.

When comparing total BF, BF of both arms, BF of right arm, BF of left arm, BF of legs, BF of right leg and BF of left leg of T1DE, T2DE and T3DE groups of different years of diving experience, no significant differences between groups were reported (Figures 4A–G).

**Figure 5:**
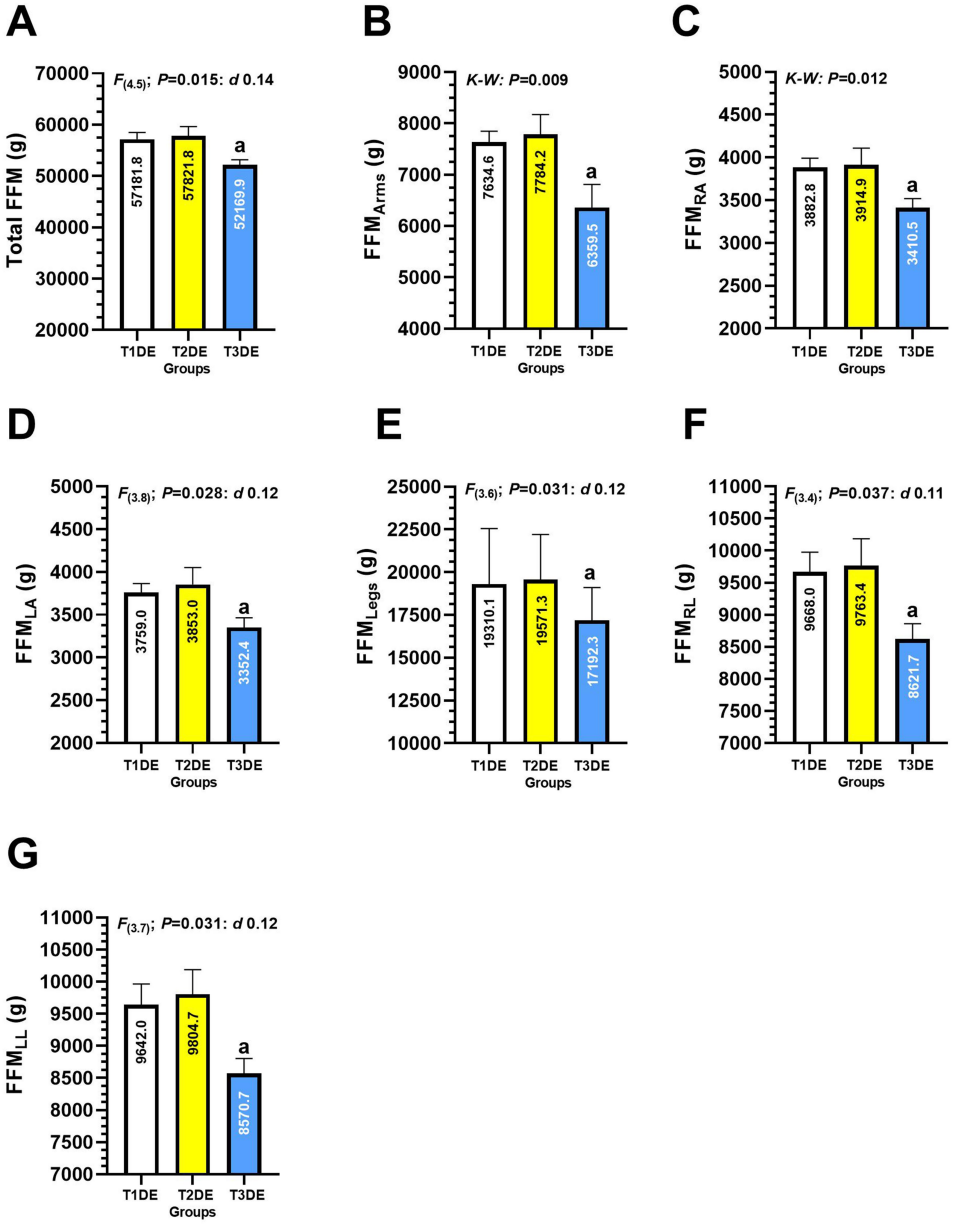
Total fat-free mass **(A)**, arms **(B)**, right arm **(C)**, left arm **(D)**, legs **(E)**, right leg **(F)**, and fat-free mass of the left leg **(G)** in Latin American divers. Groups are described as: (T1DE) Tertile 1 of diving experience (1–20 years diving), (T2DE) Tertile 2 of diving experience (21–35 years diving), and (T3DE) Tertile 3 of diving experience (36–45 years diving). Outcomes are described as: (FFM_Arms_) Fat-free mass of the arms, (FFM_RA_) Fat-free mass of the right arm, (FFM_LA_) Fat-free mass of the left arm, (FFM_Legs_) Fat-free mass of the legs, (FFM_RL_) Fat-free mass of the right leg, (FFM_LL_) Fat-free mass of the left leg. (K-W) Denotes data analyzed by Kruscal-Wallis non-parametric test at *p* < 0.05 level. (a) Denotes significant differences between group T3DE vs. T1DE at *p* < 0.05 level.

Significant differences were comparing T3DE vs. T1DE group in the outcomes total FFM (52169.9 ± 4138.0 *vs*. 57181.8 ± 6805.0 g, *p* = 0.015, *d* 0.14) (Figure 5A), FFM of arms (6359.5 ± 1851.0 *vs*. 7634.6 ± 1117.0 g, *p* = 0.009) (Figure 5B), FFM of right arm (3410.5 ± 430.8 *vs*. 3882.8 ± 569.2 g, *p* = 0.012) (Figure 5C panel **C**), FFM of left arm (3352.4 ± 463.1 vs. 3759.0 ± 546.4 g, *p* = 0.028, *d* 0.12) (Figure 5D), FFM of the legs (17192.3 ± 1919.0 *vs*. 19310.1 ± 3249.0 g, *p* = 0.031, *d* 0.12) (Figure 5E), FFM of right leg (8621.7 ± 993.5 *vs*. 9668.0 ± 1591.0 g, *p* = 0.037, *d* 0.11) (Figure 5F) and FFM of left leg (8570.7 ± 965.2 *vs*. 9642.0 ± 1681.0 g, *p* = 0.031, *d* 0.12) (Figure 5G).

### Associations between handgrip strength and bone mineral density

**Figure 6:**
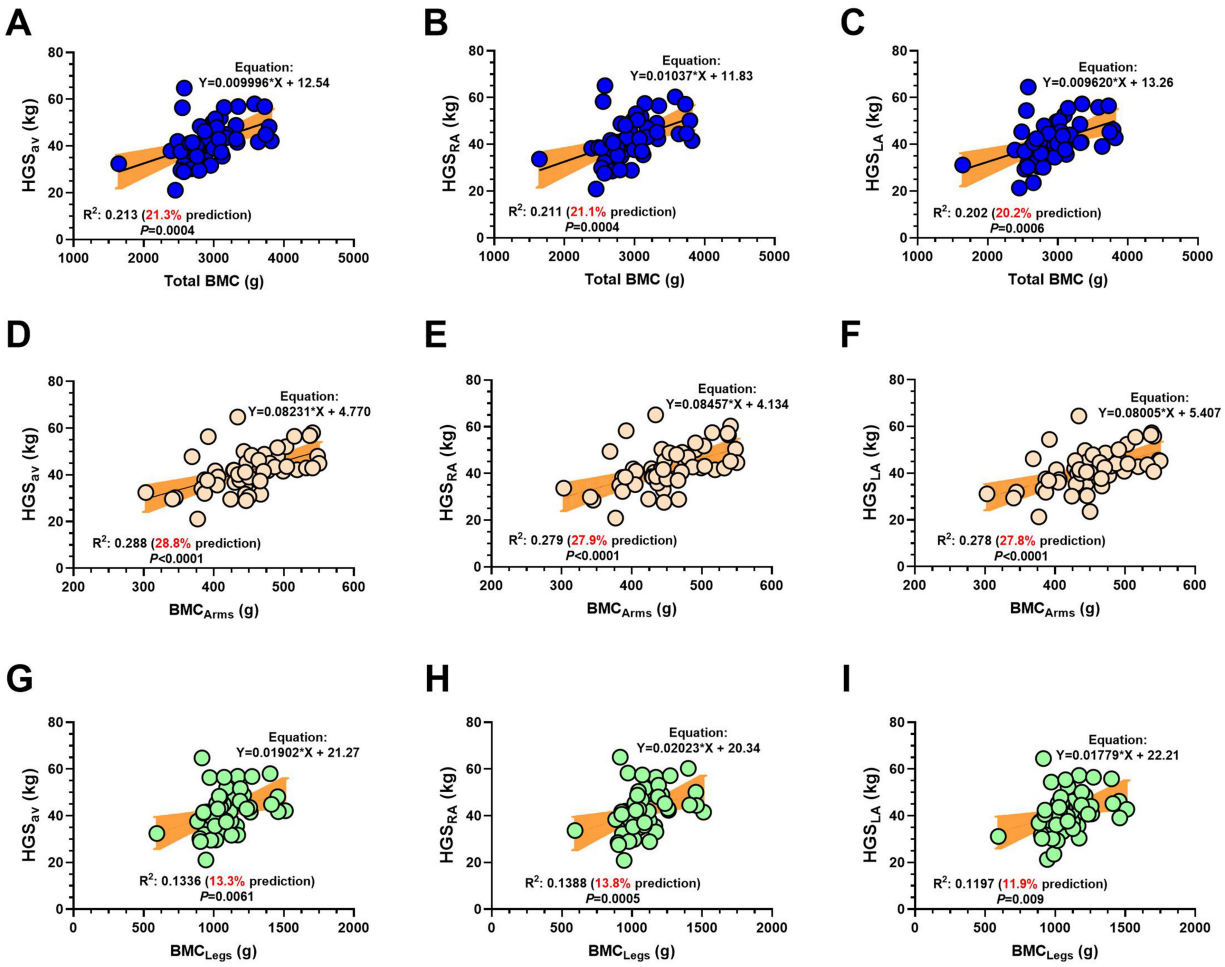
Association between handgrip strength average of both arms **(A,D,G)**, handgrip strength of the right arm **(B,E,H)** and handgrip strength of the left arm **(C,F,I)** with total bone mineral content **(A–C)**, bone mineral content of the arms **(D–F)** and bone mineral content of the legs **(G–I)** in adult Latin American diver workers. (HGS) Handgrip strength. (HGS_av_) Handgrip strength average of both arms. (BMC) Bone mineral content. (*R*^2^) Coefficient of determination, variability, and prediction. Values in red denote the % prediction of BMC concerning the HGS variable.

There was a significant correlation between HGS_av_ and Total BMC (*R*^2^ = 0.213, predictive value; 21.3% of variance explained by total BMC; Regression equation: Y = 0.00996*X + 12.54 and *p* = 0.0004) (Figure 6A). There was a significant correlation between HGS right arm and the Total BMC variable (*R*^2^ = 0.211, predictive value; 21.1% of the variance; Regression equation: Y = 0.01037*X + 11.83, *p* = 0.0004) (Figure 6B). There was a significant correlation between HGS left arm and the Total BMC variable (*R*^2^ = 0.202, predictive value; 20.2% of variance; Regression equation: Y = 0.00962*X + 13.26, *p* = 0.0006) (Figure 6C).

There was a significant correlation between HGS_av_ and BMC arms (*R*^2^ = 0.288, predictive value; 28.8% of variance explained; Regression equation: Y = 0.08231*X + 4.770, *p* < 0.0001) (Figure 6D). There was a significant correlation between HGS right arm BMC of the arms (R^2^ = 0.279, predictive value; 27.9% of variance explained; Regression equation: Y = 0.08457*X + 4.134, *p* < 0.0001) (Figure 6E). There was a significant correlation between HGS left arm and the variable BMC of the arms [*R*^2^ = 0.278 (27.8% predictive; Regression equation: Y = 0.08005*X + 5.407, *p* < 0.0001)] (Figure 6F).

There was a significant correlation between average HGS_av_ and BMC of the legs *R*^2^ = 0.1336, predictive value; 13.3% prediction; Regression equation: Y = 0.01902*X + 21.27, *p* = 0.0061, (Figure 6G). There was a significant correlation between HGS right arm and BMC of the legs (R^2^ = 0.1388, predictive value: 13.8%; Regression equation: Y = 0.0223*X + 20.34, *p* = 0.0005) (Figure 6H). Finally, there was a significant correlation between HGS left arm and BMC of the legs (*R*^2^ = 0.1197, predictive value: 11.9%), with regression equation: Y = 0.01797*X + 22.21, *p* = 0.009 (Figure 6I).

## Discussion

The objective of the present study was to describe the BMC and body composition of diver workers of different years of diving experience. A second objective was to associate the level of BMC with HGS. A second objective was to analyze the association between handgrip strength and BMC in these workers. The main results of the present study indicate that (i) there is a reduced level of BMC and FFM in the right and left leg in diver workers who have more years of diving experience (T3DE) compared to peers with less years of diving experience (T1DE) (Figure 2), (ii) there were significant correlations between HGS_av_, and HGS of the left and right arm with total BMC (~28%) and BMC of the upper body (~20%) relative to BMC of the lower body (~13%) of diver workers. Correlations are observably stronger in the arms than in the legs, and HGS_av_ was reported to have a higher predictive ability in *R*^2^ on total and regional BMC (Figure 3).

Regarding our first finding, we reported reduced BMC and FFM. A previous study used iDXA analyses in Korean female divers reported that BMC was lower proportionally to higher pressure and depth of dive, correlating with BMC and longer exposure time to water (21). In long-lived post-menopausal women divers ~72 years old, Seo et al. (34) reported that female divers did not show different BMC (measured by iDXA) compared to non-diving control pairs, however, when they experienced some type of osteoporotic spinal fracture, the divers were shown to develop compensatory mechanisms in some bone anatomical regions of the skeleton such as increased cervical lordosis and pelvic tilt relative to controls. The authors concluded that the adaptive phenomenon at the bone level was explained by the superior strength of the back muscles and the spinal mobility of the diving condition.

A study (35) that involved three groups—sprint runners, distance swimmers, and divers— reported that over 8 months of monitoring, the divers increased their body weight, without modifying their BMC or FFM. In this context, considering our results, we believe that even though the T3DE group is the group with more years of experience in diving (41.5 years) and therefore also more older (64.2 years) (Table 1) it could be considered physiologically acceptable to present a low BMC as indicated by epidemiological studies in the adult population (2, 4), however, diving activity reports an early reduction of BMC in both legs and in an isolated way in the BMC of the right and left leg (Figures 4E–G) but in parallel also presents a reduction of the FFM in the right and left leg (Figures 4A–G), putting at risk not only their capacity of muscle strength during diving tasks, but also this reduced BMC and muscle tissue increases the musculoskeletal frailty of the diver workers in daily life actions. Regarding the reduced FFM, considering that the rate of BMC loss or reduction is slower compared to changes in muscle or fat tissue, it is also consistent that divers with more diving experience, along with reporting lower BMC in legs, also present a low amount of FFM. The relevance of maintaining proper muscle mass lies in its role in maintaining appropriate levels of strength and metabolic control. For example, the muscle mass of an adult subject can uptake about 80% of the total carbohydrate (i.e., insulin-stimulated pathway) consumed by the 75 g glucose tolerance test (36). Therefore, a lower FFM reduces glucose control and increases the risk of type 2 diabetes mellitus.

The present study also found differences between groups of younger and more experienced divers in variables such as basal metabolic rate, Ruffier index, sedentary time, and HGS. Studies in young divers ~24–33 years have shown a basal metabolic rate of 2,115 Kcal/day, these values being higher than our study (range 1,403–1,468 Kcal/day), however our three groups with different years of diving experience were older (46–64 years), it being known that with age the metabolism is reduced under basal conditions (37). Conversely, the general physical capacity and cardiovascular recovery after an effort such as the Ruffier test, which on average was categorized as “Good” across the three groups of diving experience, however, there were differences in the proportions between groups, where the subjects of the T3DE group reported a smaller number of subjects in “Excellent” condition and a larger number in the “Medium” physical capacity category (Table 1). Furthermore, the time spent on sedentary activities was higher between the T3DE *vs*. T2DE groups (Table 1), where we speculate that, apart from the physiological implications in the slowing of metabolism at rest, when aging is combined with greater sedentary activity, the risk of suffering from cardiovascular and metabolic diseases is exaggeratedly increased. Similarly, it is logical to speculate that part of the low levels of HGS_av_ in the T3DE group among younger subjects and years of diving could very well be attributed to the longer time of sedentary lifestyle, much more than by the age factor, with lifestyle playing a greater role than the biological factor.

At the metabolic level (37) reported in divers ~30 years old, in overweight (BMI 25 to 30 kg/m^2^) that diving at 6 m of depth but at a T° of −5°C increased body thermogenesis, translating into a reduction in skin T° from 36.8 to 28.5°C but at the same time increasing energy expenditure from 1.9 to 2.8 Kcal/min, resulting in 6 h of diving an average metabolic utilization of 57% carbohydrates (0.40 g/min), and 42.5% fat utilization (0.13 g/min) and a higher heart rate compared to when donning equipment before diving (6.7 versus 4.3 mL/kg/min).

Regarding our third result, previous studies have reported the association between HGS_av_ and BMC in both children and adolescents (38) and adults (39). In brief, Saraiva et al. (38) reported in (*n* = 243) children and adolescents aged 4–15 years, in girls, HGS was associated with BMC of the arms, legs, trunk, spine, and total BMC of girls and boys. Thus, previous studies in both children and adult populations assess the predictive capacity of muscle strength assessed by the HGS_av_ test on BMC; however, the present study adds this additional knowledge in the present sample of Chilean diver workers. Interestingly, the reduction in BMC in divers, particularly in those with more years of diving experience, as in the T3DE group, would have a similar physiological effect in relation to life conditions without gravity, as is the case with astronauts (20). For example, space travel has been reported to cause a loss of BMC, especially in the lower body (pelvis, lumbar vertebrae, and femoral head). Part of the mechanisms attributable to this bone effect in astronauts has been reported in altered calcium metabolism, increasing the stimulation of calcium excretion through urine and a reduced intestinal calcium reabsorption (20), although in summary, long-duration space travel has negative effects on astronauts’ musculoskeletal system (20). We believe that, in the case of diver workers, considering this knowledge, more public policies should be promoted to protect muscle and bone health, especially for those with more years of diving experience, as they are at greater risk of fragility and cardiovascular disease, as can be seen from (Table 1) where in average the systolic blood pressure of the three groups are ~140 mmHg.

In secondary results, the high values of systolic blood pressure 139 to 147 mmHg and diastolic (86–9 mmHg) in the three groups of diving experience categorize the groups into high blood pressure and arterial hypertension (Table 1), which is a worrying way. Previous reports about the lifestyle of Chilean divers have already warned about the lifestyle of these workers, which includes unhealthy habits (24). Therefore, ruling out cardiovascular deterioration due to aging and promoting a much healthier lifestyle among workers in these contexts would reduce the cardiovascular and metabolic risk of these people, despite the parallel physiological risk of a reduced BMC generated by the work of diving.

In addition, due to the increased prevalence of osteoporosis and osteopenia (1, 2), and that a low BMC is a precursor to a diagnosis of osteoporosis (2), and that previous Chilean studies revealed a prevalence of frailty in men 35.1% (i.e., frailty as defined by Fried’s Phenotype Scale) (4), there is little information regarding some physiological and environmental conditions of some employment conditions such as diver workers dedicated to mollusks extraction in the coast. Moreover, to prevent risk factors for frailty, there is a need to look for predicting early a frailty condition and BMC deterioration, such as the previous reports of energy expenditure (17), and muscle size for these aims (18, 19). Here we added more information about the utility of HGS associated with BMF and the fat-free mass in diver workers, and future studies could continue exploring in larger sample sizes and by cross-sectional and longitudinal studies the HGS capacity for predicting BMC and body composition in diver workers. Potential future hypothesis of interest to solve could include whether other physical fitness variables in both young and more older diver workers (i.e., cardiorespiratory or other muscle strength outcomes) are associated and predict BMC in divers with history of bone accident, and include not only diver workers of the aquiculture/fishing area but also other types of diving specialties such as rescue and deeper diving with different lifestyle.

### Limitations and strengths

This study is not without its weaknesses, for example, (i) only people who attended voluntarily were assessed and not the total number of active member subjects of each social group invited from the cities involved, (ii) the heart rate was recorded manually by taking heart rate per minute during the Ruffier test, however, this parameter was obtained from an exercise specialist in the diving area, (iii) the seasons with longer or shorter diving times were not considered, nor was the temperature of the water in which they dived, and (iv) the sample size was statistically sufficient but future studies could increase the sample size amount to increase the statistical power.

However, it also has some strengths; (i) the measurements were taken in a population that is not usually accessible and that usually does not attend preventive health checks at health system, (ii) the body composition analyses were carried out using the iDXA equipment, which is gold standard for the total and segmental analysis of BF, FFM and BMC, and (iii) the measurements taken are preventive, which will allow future health promotion actions to be proposed for the participants in this study.

## Conclusion

Diver workers with more years of experience have a reduced level of BMC and FFM in the legs compared to peers with less experience, and HGS shows a significant association with BMC. These results suggest the need for future longitudinal studies to monitor health in diver workers based on robust iDXA body composition analyses to confirm our findings and include other physical fitness parameters in different diving specialties.

## Data Availability

The data presented in the study are deposited in the https://figshare.com repository, accession link: https://figshare.com/articles/dataset/Database_Divers/28914317?file=54128975.
