# Supplementary material for: Bone mineral content determined by energy X-ray absorptiometry correlates with handgrip strength in Latin American divers
Source: Front Public Health. 2025 Jul 14;13:1591242. doi: 10.3389/fpubh.2025.1591242 (PMC12301340; doi:10.3389/fpubh.2025.1591242)
Supplement: Supplementary file 1 [file Table_1.docx]

Supplementary Material 1

# Supplementary Material.

| **Supplementary Material.** Normality analyses using Shapiro-Wilk test by each tertile group on main or secondary outcomes. | | | | |
| --- | --- | --- | --- | --- |
| **Main/Secondary outcome** |  | **T1DE**  **(W; *P*value)** | **T2DE**  **(W; *P*value)** | **T3DE**  **(W; *P*value)** |
| *Main outcomes* | ***Bone mineral content*** |  |  |  |
|  | Total BMC | 0.956; *P*=0.299 | 0.920; *P*=0.326 | 0.928; *P*=0.207 |
|  | BMC_Arms_ | 0.976; *P*=0.764 | 0.907; *P*=0.227 | 0.951; *P*=0.473 |
|  | BMC_RA_ | 0.970; *P*=0.616 | 0.941; *P*=0.537 | 0.938; *P*=0.300 |
|  | BMC_LA_ | 0.979; *P*=0.846 | 0.889; *P*=0.184 | 0.975; *P*=0.911 |
|  | BMC_Legs_ | 0.970; *P*=0.614 | 0.909; *P*=0.240 | 0.977; *P*=0.935 |
|  | BMC_RL_ | 0.971; *P*=0.637 | **0.836; *P*=0.002** | 0.965; *P*=0.736 |
|  | BMC_LL_ | 0.962; *P*=0.419 | 0.967; *P*=0.860 | 0.968; *P*=0.798 |
| *Main outcomes* | ***Body fat*** |  |  |  |
|  | Total BF | **0.469; *P*<0.0001** | 0.956; *P*=0.722 | 0.969; *P*=0.809 |
|  | BF_Arms_ | 0.976; *P*=0.764 | 0.907; *P*=0.227 | 0.951; *P*=0.473 |
|  | BF_RA_ | 0.970; *P*=0.616 | 0.941; *P*=0.537 | 0.938; *P*=0.300 |
|  | BF_LA_ | 0.979; *P*=0.846; | 0.899; *P*=0.184 | 0.975; *P*=0.911 |
|  | BF_Legs_ | 0.970; *P*=0.614 | 0.909; *P*=0.240 | 0.977; *P*=0.935 |
|  | BF_RL_ | 0.971; *P*=0.637 | **0.836; *P*=0.0002** | 0.965; *P*=0.736 |
|  | BF_LL_ | 0.965; *P*=0.736 | 0.962; *P*=0.419 | 0.967; *P*=0.860 |
| *Main outcomes* | ***Fat-free-mass*** |  |  |  |
|  | Total FFM | 0.973; *P*=0.684 | **0.724; *P*<0.001** | 0.943; *P*=0.392 |
|  | FFM_Arms_ | 0.978; *P*=0.815 | **0.721; *P*=0.0009** | 0.943; *P*=0.392 |
|  | FFM_RA_ | 0.973; *P*=0.684 | **0.724; *P*=0.001** | 0.943; *P*=0.392 |
|  | FFM_LA_ | 0.975; *P*=0.744 | **0.757; *P*=0.002** | 0.935; *P*=0.265 |
|  | FFM_Legs_ | 0.951; *P*=0.228 | 0.907; *P*=0.227 | 0.944; *P*=0.373 |
|  | FFM_RL_ | 0.962; *P*=0.411 | 0.913; *P*=0.267 | 0.984; *P*=0.998 |
|  | FFM_LL_ | 0.963; *P*=0.445 | **0.856; *P*=0.005** | 0.942; *P*=0.351 |
| *Main outcomes* | HGS_av_ (kg) | 0.974; *P*=0.713 | 0.966; *P*=0.846 | 0.919; *P*=0.143 |
| Secondary outcomes | Age (y) | 0.961; *P*=0.392 | 0.924; *P*=0.358 | 0.952; *P*=0.503 |
|  | Diving experience (y) | **0.858; *P*=0.001** | **0.724; *P*=0.001** | **0.791; *P*=0.0001** |
| Secondary outcomes | ***Anthropometric*** |  |  |  |
|  | Height (m) | 0.955; *P*=0.287 | 0.909; *P*=0.240 | 0.969; *P*=0.809 |
|  | Weight (kg) | 0.975; *P*=0.751 | 0.903; *P*=0.205 | 0.973; *P*=0.876 |
|  | BMI (kg·m^2^) | 0.956; *P*=0.302 | 0.977; *P*=0.950 | 0.962; *P*=0.679 |
| Secondary outcomes | ***Arterial hypertension*** |  |  |  |
|  | Systolic blood pressure (mmHg) | **0.918; *P*=0.036** | 0.937; *P*=0.486 | 0.937; *P*=0.294 |
|  | Diastolic blood pressure (mmHg) | 0.970; *P*=0.601 | 0.867; *P*=0.072 | 0.943; *P*=0.365 |
|  | Heart rate rest (beats/min) | **0.905; *P*=0.017** | 0.928; *P*=0.397 | 0.928; *P*=0.211 |
|  | BMR (kcal/kg) | 0.954; 0.291 | 0.861; 0.060 | 0.941; 0.337 |
| Secondary outcomes | ***Physical fitness condition*** |  |  |  |
|  | *Ruffier index* | 0.960; *P*=0.385 | 0.961; *P*=0.785 | 0.986; *P*=0.058 |
| Secondary outcomes | ***PA patterns (GPAQ)*** |  |  |  |
|  | PAVI (d/week) | **0.831; *P*=0.0005** | 0.936; *P*=0.476 | **0.818; *P*=0.003** |
|  | PAMI (d/week) | **0.841; *P*=0.0008** | **0.740; *P*<0.001** | 0.905; *P*=0.083 |
|  | PALI (d/week) | **0.802; *P*=0.0002** | 0.905; *P*=0.217 | **0.858; *P*=0.014** |
|  | Total PA (min/wk) | 0.935; *P*=0.095 | 0.957; *P*=0.745 | 0.917; *P*=0.131 |
|  | Total PA (MET/wk) | **0.831; *P*=0.0005** | 0.936; *P*=0.476 | **0.818; *P*=0.0003** |
|  | Total sedentary time (min/wk) | **0.906; *P*=0.019** | 0.892; *P*=0.150 | **0.771; *P*=0.0008** |
| **Groups are described as**; (T1DE) Tertile 1 of diving experience (1-20 years diving), (T2DE) Tertile 2 of diving experience (21-35 years diving) and (T3DE) Tertile 3 of diving experience (36-45 years diving). **Outcomes are described as;** (HGS_av_) Handgrip muscle strength average of both arms. (PA) Physical activity. (PAVI) Physical activity of vigorous intensity, (PAMI) Physical activity of moderate intensity, (PALI) Physical activity of light intensity. (MET) Metabolic equivalent of task in resting. (BMI) Body mass index. (BMR) Basal metabolic rate. Bold values denote variables with no normal distribution. | | | | |
